# Differential response to neoadjuvant endocrine therapy for Black/African American and White women in NCDB

**DOI:** 10.1007/s10549-023-07106-8

**Published:** 2023-09-23

**Authors:** Veronica Jones, Mary C. Schroeder, Mya L. Roberson, James De Andrade, Ingrid M. Lizarraga

**Affiliations:** 1 Department of Surgery, City of Hope National Medical Center, 1500 E Duarte Rd, Duarte, CA 91010 USA; 2 Division of Health Services Research, University of Iowa College of Pharmacy, 180 S Grand Ave, Iowa City, IA 52242 USA; 3 Department of Health Policy and Management, UNC Gillings School of Global Public Health, 135 Dauer Drive, Chapel Hill, NC 27599 USA; 4 Department of Surgery, University of Iowa Roy J. and Lucille A. Carver College of Medicine, 200 Hawkins Drive, Iowa City, IA 52242 USA

**Keywords:** Endocrine therapy, National Cancer Database, Resistance

## Abstract

**Purpose:**

Compared to White women, there are higher mortality rates in Black/African American (BAA) women with hormone receptor-positive breast cancer (HR + BC) which may be partially due to differences in treatment resistance. We assessed factors associated with response to neoadjuvant endocrine therapy (NET).

**Methods:**

The National Cancer Database (NCDB) was queried for women with clinical stage I–III HR + BC diagnosed 2006–2017 and treated with NET. Univariate and multivariate analyses described associations between the sample, duration of NET, and subsequent treatment response, defined by changes between clinical and pathological staging.

**Results:**

The analytic sample included 9864 White and 1090 BAA women. Compared to White women, BAA women were younger, had more co-morbidities, were higher stage at presentation, and more likely to have > 24 weeks of NET. After excluding those with unknown pT/N/M, 3521 White and 365 BAA women were evaluated for NET response. On multivariate analyses, controlling for age, stage, histology, HR positivity, and duration of NET, BAA women were more likely to downstage to pT0/Tis (OR 3.0, CI 1.2–7.1) and upstage to Stage IV (OR 2.4, CI 1.002–5.6). None of the women downstaged to pT0/Tis presented with clinical stage III disease; only 2 of the women upstaged to Stage IV disease presented with clinical Stage I disease.

**Conclusion:**

Independent of NET duration and clinical stage at presentation, BAA women were more likely to experience both complete tumor response and progression to metastatic disease. These results suggest significant heterogeneity in tumor biology and warrant a more nuanced therapeutic approach to HR + BC.

## Introduction

When compared to non-Hispanic White women, Black/African American (BAA) women with breast cancer have 40% higher mortality [1]. While the higher incidence of triple-negative breast cancer among BAA women is a known contributor to the disparity, BAA women (vs. NHW) also die at a higher rate from hormone receptor-positive (HR+) breast cancer [1]. In fact, the mortality rate from HR+ breast cancer is twice as higher in BAA women than it is in NHW women [2, 3]. Factors contributing to this disparity are complex. Race encompasses a multitude of factors, including one’s lived experience as well as biological factors, such as ancestry. To date, studies investigating drivers of racial HR+ breast cancer mortality disparities have examined varying social determinants of health; the mortality difference persists when controlling for stage at presentation, tumor grade, and treatment. The cancer biology itself must also be examined when dissecting drivers of outcomes.

HR+ breast cancer is highly heterogenous; it can be subdivided into the classifications of luminal A and luminal B. While luminal A breast cancer is estrogen and progesterone receptor positive with HER2 receptor negativity and low proliferation index as measured by Ki67, luminal B breast cancer often has lower hormone receptor sensitivity, higher proliferation index and can be either HER2 positive or negative. As a result, luminal B breast cancer recurs more often and earlier, and is associated with worse prognosis. It is more endocrine resistant than luminal A breast cancer but also responds less than triple-negative or HER2-enriched subtypes to chemotherapy [4]. Black women with luminal B breast cancer also have gene expression patterns that share similarities to basal-like tumors [5].

Despite the heterogeneity in presentation, HER2 status, and outcome, HR+ breast cancer is treated with relative uniformity; all patients receive endocrine therapy at some point in their management. In Stage I–III HR+ breast cancer, this is most often in the adjuvant setting once measurable disease has been resected. Endocrine resistance is only identified at the time of relapse. Contrary to this, administration of endocrine therapy in the neoadjuvant setting allows measure of resistance with disease in place. While this is not standard practice, it has been utilized in several settings: (1) clinical trials [6], (2) when a patient has significant co-morbidities, (3) as an attempt to downstage the tumor when chemotherapy is thought to be ineffective (such as in the case of a low proliferation index) [7], or (4) more recently in the Covid era as a temporizing measure to surgery [8]. Typically, treatment for 3–6 months is felt to be sufficient to measurable durable response [9]. However, neoadjuvant administration of endocrine therapy also provides an opportunity to evaluate endocrine sensitivity. This may prove to be a means to define HR+ breast cancer further and determine how therapy resistance perpetuates the mortality disparity.

The purpose of this study was to examine whether duration of treatment and/or response to neoadjuvant endocrine therapy (NET) might differ in BAA and White women. We hypothesized that there would be shorter duration or diminished response to endocrine therapy in the neoadjuvant setting among BAA women with HR+ breast cancer given the witnessed disparity in histology and mortality.

## Materials and methods

The National Cancer Database (NCDB) was queried for women with American Joint Committee on Cancer (AJCC) 7th edition clinical Stage I–III, microscopically confirmed, HR+ breast cancer treated between the years 2006 and 2017 with endocrine therapy prior to surgical resection (i.e., NET). Single-hormone receptor (estrogen or progesterone) positivity was allowed. HER2 status was not available for all patients and was, therefore, not defined as part of the inclusion criteria. Patients receiving neoadjuvant chemotherapy and those with unknown clinical TNM staging were excluded from the analysis.

Patient demographics, tumor characteristics, and treatment data were collected. Race was recorded in NCDB by either self-report or as determined by the treating provider. Tumors were categorized using ICD-O-3 histology codes as ductal only (8500), lobular only (8520), ductal and lobular (8522), and other (8010, 8050, 8140, 8201, 8211, 8255, 8480, 8507, 8523, 8524). Duration of NET was defined as the difference between the date of initiation of NET and date of first operation, as exact duration was not recorded in NCDB. Positivity in both ER and PR (versus single receptor) was noted, as well as HER2 status.

The reporting facilities were categorized as Community Cancer Programs, Comprehensive Community Cancer Programs, Academic/Research Programs (includes NCI-designated comprehensive cancer centers), and Integrated Network Cancer Programs. Socioeconomic measures were also collected, including primary payor, rurality of patient’s county of residence (defined using Rural–Urban Continuum Codes), and variables measured at the patient’s residential ZIP-code level (median household income, percent not graduating from high school).

Known pathologic stage information was required for outcome measures of response to therapy. Downstaging was defined as a pathologic stage being lower than the clinical stage and measured for the stage group, as well as T and N stages individually. Upstaging was defined as pathologic stage being higher than the clinical stage and measured for the stage group, as well as T, N, and M stages individually. The primary endpoints included tumor (T) upstaging and downstaging, as well as downstaging to pT0/is and upstaging to Stage IV disease (pM1). Univariate analyses were used to describe the study sample and assess associations between the outcomes and race, duration of NET, and clinical characteristics. Multivariate logistic regressions were performed to examine differences across outcomes by race, controlling for duration of NET, age, both ER and PR positive, clinical stage, and histology.

Statistical significance was set at 5% and all tests were two tailed. The study sample was generated using SAS software, Version 9.4 (SAS Institute Inc., Cary, NC). Statistical analyses were performed using Stata Statistical Software: Release 15 (StataCorp LLC, College Station, TX). This project was reviewed and approved for analysis by NCDB through Institutional Review Board agreements.

The NCDB is a joint project between the Commission on Cancer of the American College of Surgeons and the American Cancer Society. The data in this study are derived from a de-identified NCDB file under a Data Use Agreement. The American College of Surgeons and the Commission on Cancer have not verified and are not responsible for the analytic or statistical methodology employed, or the conclusions drawn from these data by the investigators.

## Results

**Fig. 1:**
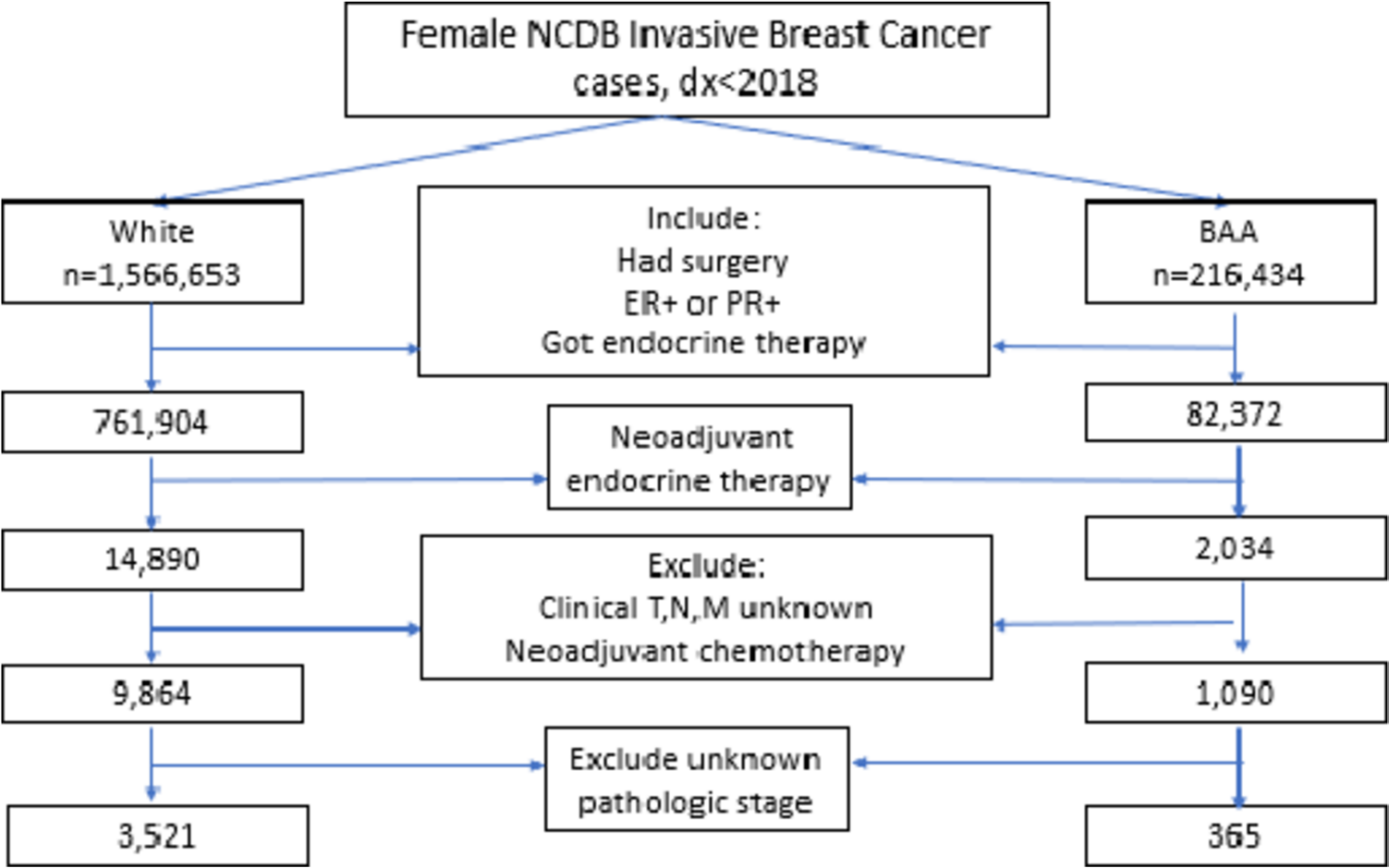
Inclusion and exclusion criteria for study cohort (2006–2017). After meeting all inclusion and exclusion criteria, the final sample included 9864 White and 1090 BAA women

The initial sample of BAA or White women with breast cancer diagnosed 2006–2017 with recorded breast cancer histologies included 1,566,653 White women and 216,434 BAA women. Only women with clinical Stage I–III estrogen or progesterone receptor (hormone receptor/HR)-positive breast cancer who received neoadjuvant endocrine therapy followed by surgery were included. The number of BAA women included dropped more substantially due to a higher number having HR disease (30.5% of the BAA cohort compared to 16.6% of White women). Women with unknown clinical staging or who had received neoadjuvant chemotherapy were then excluded. While women with HER2 positivity were not excluded, the vast majority of the women were HER2 negative. There was no significant difference between the two groups in HER2 status. BAA women were, however, disproportionately excluded due to a higher percentage receiving neoadjuvant chemotherapy (42.9% of BAA women compared to 29.9% of White women). The final sample included 9864 White and 1090 BAA women (Fig. 1). Of these, 8036 White and 883 BAA women were known HER2 negative.

The median age at diagnosis was slightly lower for BAA (66.5 years) than for White women (68 years). Co-morbidity scores were higher in the BAA group (79.6% of White women with Charlson–Deyo score 0 compared to 70.9% of Black women). BAA women were also more likely to have poorly differentiated tumors and more likely to have single-hormone receptor positivity (Table 1).

**Table 1:** Clinical and patient characteristics of study sample

	White		BAA		*p* value*
	*N*	%	*N*	%	
Sample size	9864		1090		
Median (IQR) age at diagnosis	68	(18)	66.5	(17)	0.019
Median (IQR) duration of NET**	115	(135)	129	(147)	< 0.001
Duration of NET					
Up to 8 weeks	2964	30	275	25.2	< 0.001
> 8 to 24 weeks	4087	41.4	431	39.5	
> 24 weeks	2813	28.5	384	35.2	
Charlson–Deyo Score					
0	7852	79.6	773	70.9	< 0.001
1	1396	14.2	207	19.0	
2	411	4.2	66	6.1	
3+	205	2.1	44	4.0	
Grade					
Well differentiated	2813	28.5	258	23.7	< 0.001
Moderately differentiated	5255	53.3	539	49.4	
Poorly or undifferentiated	1308	13.3	216	19.8	
Unknown	488	4.9	77	7.1	
Histology					
Ductal only (8500)	6220	63.1	685	62.8	< 0.001
Lobular only (8520)	807	8.2	70	6.4	
Ductal and lobular (8522)	1993	20.2	200	18.3	
Other	844	8.6	135	12.4	
Both ER and PR positive					
No	2356	23.9	291	26.7	0.045
Yes	7472	75.8	798	73.2	
Unknown	36	0.4	1	0.1	
HER2 positive					
No	8036	81.5	883	81	0.737
Yes	317	3.2	32	2.9	
Unknown	1511	15.3	175	16.1	
Clinical stage group					
I	3015	30.6	277	25.4	< 0.001
II	5420	54.9	622	57.1	
III	1429	14.5	191	17.5	
Clinical stage, cT					
cT0	3	0.03	0	0	< 0.001
cT1	3252	33.0	313	28.7	
cT2	4518	45.8	505	46.3	
cT3	1251	12.7	185	17.0	
cT4	840	8.5	87	8.0	
Clinical stage, cN					
cN0	7832	79.4	763	70.0	< 0.001
cN1	1668	16.9	280	25.7	
cN2	280	2.8	36	3.3	
cN3	84	0.9	11	1.0	
Pathologic stage group					
0	45	0.5	16	1.5	< 0.001
I	3108	31.5	299	27.4	
II	3905	39.6	432	39.6	
III	1696	17.2	235	21.6	
IV	27	0.3	6	0.6	
Unknown	1083	11.0	102	9.4	
Pathologic stage, pT					
PT0is	123	1.2	23	2.1	0.029
pT1	4129	41.9	421	38.6	
pT2	3672	37.2	409	37.5	
pT3	926	9.4	125	11.5	
pT4	369	3.7	42	3.9	
pTX	645	6.5	70	6.4	
Pathologic stage, pN					
pN0	4983	50.5	512	47	0.006
pN1	2460	24.9	289	26.5	
pN2	733	7.4	106	9.7	
pN3	409	4.1	57	5.2	
pNX	1279	13	126	11.6	
Pathologic stage, pM					
pM0	3778	38.3	382	35.0	0.025
pM1	30	0.3	7	0.6	
pMX	6056	61.4	701	64.3	
Reporting facility category					
CCP	550	5.6	48	4.4	< 0.001
CCCP	3645	37.0	308	28.3	
ACAD + NCIP	3224	32.7	498	45.7	
INCP	2334	23.7	220	20.2	
Unknown	111	1.1	16	1.5	
Primary payor					
Not insured	169	1.7	37	3.4	< 0.001
Private/managed care	3568	36.2	313	28.7	
Medicaid	510	5.2	139	12.8	
Medicare	5406	54.8	575	52.8	
Other govt	74	0.8	9	0.8	
Unknown	137	1.4	17	1.6	
RUCC (patient county)					
Metro 1 million+	5469	55.4	800	73.4	< 0.001
Metro 0.25–1 million	1983	20.1	156	14.3	
Metro < 0.25 million	697	7.1	41	3.8	
Urban 20,000+	496	5	27	2.5	
Urban < 20,000 or rural	911	9.2	43	3.9	
Unknown	308	3.1	23	2.1	
Median household income (patient ZIP)					
< $38,000	1175	11.9	368	33.8	< 0.001
$38,000–$47,999	1987	20.1	203	18.6	
$48,000–$62,999	2348	23.8	199	18.3	
$63,000+	3147	31.9	176	16.1	
Unknown	1207	12.2	144	13.2	
Percent not graduate HS (patient ZIP)					
< 7%	2651	26.9	116	10.6	< 0.001
7–12.9%	2898	29.4	227	20.8	
13–20.9%	1910	19.4	333	30.6	
21%+	1204	12.2	271	24.9	
Unknown	1201	12.2	143	13.1	

The median age at diagnosis was slightly lower for BAA (66.5) than for White women (68 years). Co-morbidity scores were higher in the BAA group (79.6% of White women with Charlson–Deyo score 0 compared to 70.9% of Black women). BAA women were also more likely to have poorly differentiated tumors and more likely to have single-hormone receptor positivity

*BAA* Black/African American, *IQR* interquartile range, *NET* neoadjuvant endocrine therapy, *ER* estrogen receptor, *PR* progesterone receptor, *HER2* human epidermal growth factor receptor 2, *CCP* Community Cancer Program, *CCCP* Comprehensive Community Cancer Program, *ACAD* + *NCIP* Academic Comprehensive Cancer Program or NCI-Designated Comprehensive Cancer Center Program, *INCP* Integrated Network Cancer Program, *RUCC* Rural–Urban Continuum Codes, *ZIP* zone improvement plan, *HS* high school

*p value from chi^2^ test (categorical variables) and Kolmogorov–Smirnov test (continuous variables)

**Measured in days

With respect to stage at presentation, BAA women were more likely to present with higher clinical tumor and nodal stage. White women had lower overall clinic stage, higher proportion of smaller tumor size (cT1), and node negative (cN0) disease. Overall White women were also more likely to have pathologic Stage I disease and no evidence of positive pathologic nodes.

Sociodemographic variables differed between the two groups with *p* < 0.001, including payor status (12.8% BAA with Medicaid vs 5.2% White), income (33.8% BAA with < $38,000 median household income for the patient ZIP vs 11.9% White), rurality of residence (73.4% BAA living in metropolitan counties with over 1 million residents vs 55.4% White), and type of treatment facility (academic center or NCI-designated comprehensive cancer center: 45.7% BAA and 32.7% White, respectively; comprehensive community cancer center: 28.3% BAA and 37.0% White, respectively, Table 1).

**Fig. 2:**
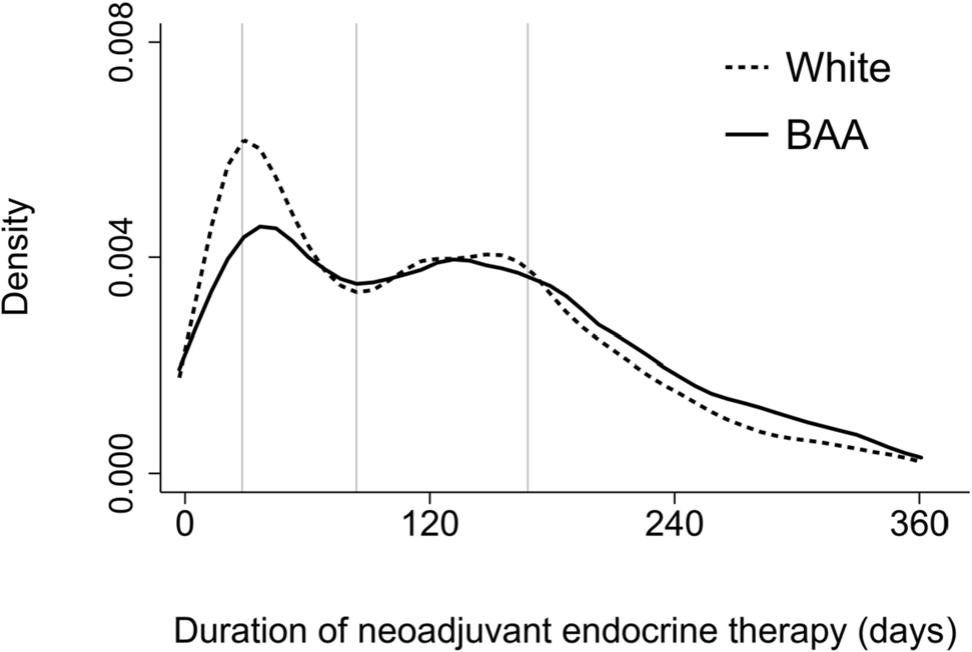
Duration of neoadjuvant endocrine therapy in days for BAA and White women. (Duration of treatment was defined as the difference in days between the start of neoadjuvant endocrine therapy and the date of definitive surgery). Median duration of NET was higher in BAA (129 days) than White women (115 days)

Duration of NET by race is plotted in Fig. 2. Median duration of NET was higher in BAA (129 days) than White women (115 days). Multiple factors were associated with duration of NET. A larger proportion of BAA women received > 24 weeks of NET than White women (36.2% vs 27.9%). Age at diagnosis was associated with duration of NET: 38.5% of women at least 80 years of age received > 24 weeks weeks of NET, compared with 17.4% of women diagnosed < 50 years (Table 2). Despite being statistically significant, there were no clear trends in duration of NET by primary payor, rurality of residence, or median household income. In contrast, duration of NET varied across reporting facility category: 32.8% of women treated at academic centers or NCI-designated comprehensive cancer centers received > 24 weeks of NET, compared with 28.3% at comprehensive community cancer programs and 18.1% at community cancer programs.

**Table 2:** Duration of neoadjuvant endocrine therapy by patient/clinical characteristics and outcomes

	Up to 8 weeks		> 8 to 24 weeks		> 24 weeks		*p* value
	*N*	Row%	*N*	Row%	*N*	Row%	
Race							
White	1114	31.6	1424	40.4	983	27.9	0.003
BAA	94	25.8	139	38.1	132	36.2	
Age at diagnosis							
< 50	196	50.9	122	31.7	67	17.4	< 0.001
50–59	291	36.4	340	42.6	168	21.0	
60–69	355	28.5	525	42.1	367	29.4	
70–79	242	25.2	396	41.2	323	33.6	
≥ 80	124	25.1	180	36.4	190	38.5	
Charlson–Deyo Score							
0	981	31.5	1243	39.9	891	28.6	0.678
1	150	30.4	204	41.4	139	28.2	
2	43	26.4	65	39.9	55	33.7	
3+	34	29.6	51	44.3	30	26.1	
Both ER and PR positive							
No	228	28.8	351	44.3	213	26.9	0.116
Yes	976	31.6	1209	39.2	899	29.2	
Unknown	4	40.0	3	30.0	3	30.0	
Clinical stage group							
I	651	52.2	435	34.9	160	12.8	
II	491	22.5	948	43.3	748	34.2	
III	66	14.6	180	39.7	207	45.7	
Reporting facility category							
CCP	64	29.0	117	52.9	40	18.1	< 0.001
CCCP	465	32.9	548	38.8	399	28.3	
ACAD + NCIP	330	24.7	567	42.5	438	32.8	
INCP	321	36.8	321	36.8	231	26.5	
Unknown	28	62.2	10	22.2	7	15.6	
Primary payor							
Not insured	13	17.3	36	48.0	26	34.7	< 0.001
Private/managed care	570	36.7	618	39.7	367	23.6	
Medicaid	58	23.4	104	41.9	86	34.7	
Medicare	550	28.3	776	39.9	617	31.8	
Other govt	9	33.3	14	51.9	4	14.8	
Unknown	8	21.1	15	39.5	15	39.5	
RUCC (patient county)							
Metro 1 million+	720	32.2	847	37.8	672	30.0	0.007
Metro 0.25–1 million	216	28.2	343	44.7	208	27.1	
Metro < 0.25 million	71	28.6	116	46.8	61	24.6	
Urban 20,000+	50	26.2	88	46.1	53	27.7	
Urban < 20,000 or rural	117	35.7	119	36.3	92	28.0	
Unknown	34	30.1	50	44.2	29	25.7	
Median household income (patient ZIP)							
< $38,000	151	29.8	212	41.8	144	28.4	< 0.001
$38,000–$47,999	232	31.3	326	44.0	183	24.7	
$48,000–$62,999	288	32.3	333	37.3	271	30.4	
$63,000+	398	33.5	436	36.7	354	29.8	
Unknown	139	24.9	256	45.9	163	29.2	
Response to NET, T/N/M stage**							
% downstage T	152	12.6%	473	30.3%	435	39.0%	
% downstaged to pT0/is	5	0.4%	10	0.6%	16	1.4%	
% downstage N	15	1.2%	44	2.8%	56	5.0%	
% upstage T	121	10.0%	163	10.4%	102	9.1%	
% upstage N	298	24.7%	447	28.6%	378	33.9%	
% upstage M	11	0.9%	12	0.8%	10	0.9%	
Response to NET, stage group**							
% downstage	112	9.3%	293	18.8%	280	25.1%	
% no change	885	73.3%	994	63.6%	639	57.3%	
% upstage	211	17.5%	276	17.7%	196	17.6%	

Multiple factors were associated with duration of NET, including Black race

*BAA* Black/African American, *ER* estrogen receptor, *PR* progesterone receptor, *HER2* human epidermal growth factor receptor 2, *CCP* Community Cancer Program, *CCCP* Comprehensive Community Cancer Program, *ACAD* + *NCIP* Academic Comprehensive Cancer Program or NCI-Designated Comprehensive Cancer Center Program, *INCP* Integrated Network Cancer Program, *RUCC* Rural–Urban Continuum Codes, *ZIP* zone improvement plan, *NET* neoadjuvant endocrine therapy

*p value from chi^2^ test

**Treatment response evaluated in sample with known pT/N/M stage (*N* = 3886)

After excluding those with unknown pT/N/M, 3521 White and 365 BAA women were evaluated for NET response. Tumor downstaging was more common than tumor upstaging, occurring in 27.3% and 9.9% of the study sample, respectively. In contrast, nodal downstaging was far less common than nodal upstaging, occurring in 3.0% and 28.9% of the study sample, respectively. Overall, 0.8% of all women downstaged to pT0/is (*N* = 31) and 0.9% upstaged to Stage IV disease (*N* = 33). No women with clinical Stage III disease were noted to downstage to pT0/is after NET. All but 2 of the women who upstaged to Stage IV were clinical Stage II/III disease at presentation. Benefit of treatment was not a simple or consistent function of duration of therapy. Longer treatment was associated with better treatment response in terms of tumor downstaging (39.0% for > 24 weeks of NET vs 12.6% for < 8 weeks of NET) and downstaging to pT0/is (1.4% for > 24 weeks of NET vs 0.4% for < 8 weeks of NET). However, with regard to nodal stage, longer treatment was associated with both nodal downstaging (5.0% for > 24 weeks of NET vs 1.2% for < 8 weeks of NET) and nodal upstaging (33.9% for > 24 weeks of NET vs 24.7% for < 8 weeks of NET) (Table 2).

**Fig. 3:**
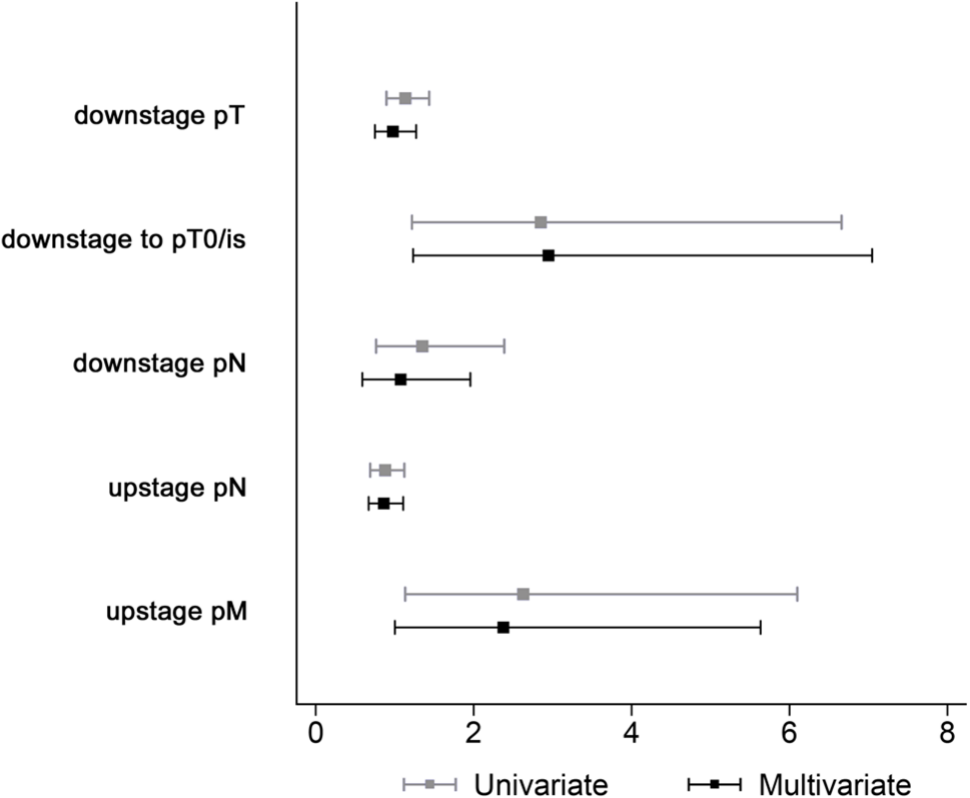
Odds of outcome for BAA compared to Whites from univariate and multivariate logistic regressions. (Multivariate models controlled for duration of neoadjuvant endocrine therapy, age, clinical stage, whether one or both hormone receptors were positive, and tumor histology). Compared with White women, BAA women were more likely to downstage to pT0/is (OR 2.9, 1.2–6.7) and more likely to upstage to Stage IV disease (OR 2.6, 1.1–6.1)

On univariate analysis, certain factors were found to contribute to tumor response (Table 3). While there was no difference across races in total tumor downstaging rates, those who received > 24 weeks of NET were more likely to downstage compared to those who received < 8 weeks of treatment (OR 3.5, CI 1.3–9.6). Adding 10 years to the age at diagnosis decreased the odds of downstaging to pT0/is by 30% (OR 0.7, CI 0.5–0.9). Clinical stage group, histology, and whether one or both hormone receptors were positive were not associated with downstage to pT0/is but were associated with tumor upstaging. Lobular breast cancers were more likely than ductal cancers to undergo tumor upstaging while on NET (OR 2.4, CI 1.7–3.4), as well as breast cancers with a mix of ductal and lobular histologies (OR 2.6, 2.1–3.4). Differential response to NET by race was assessed with univariate logistic regressions (Fig. 3). Compared with White women, BAA women were more likely to downstage to pT0/is (OR 2.9, 1.2–6.7) and more likely to upstage to Stage IV disease (OR 2.6, 1.1–6.1). This was also true when evaluating HER2-negative patients only (downstage to pT0/is OR 2.8, 1.1–7.2; upstage to Stage IV OR 3, 1.2–7.9). None of the other outcomes were statistically significant. Similar results were seen in the multivariate analyses (Fig. 3): BAA women were still more likely to downstage to pT0/is (OR 3.0, 1.2–7.1) as well as upstage to Stage IV disease (OR 2.4, 1.002–5.6), controlling for differences in duration of NET, age at diagnosis, HR positivity, clinical stage, and histology (tabular results not shown).

**Table 3:** Univariate models of response to neoadjuvant endocrine therapy by patient/tumor characteristics and duration of therapy

	Downstaged to pT0/is			Upstaged in tumor size			Upstaged to stage IV		
	OR	95% CI	*p* Value	OR	95% CI	*p* Value	OR	95% CI	*p* Value
Race									
White	Ref			Ref			Ref		
BAA	2.85	1.22–6.66	0.016	1.06	0.74–1.51	0.749	2.63	1.13–6.10	0.024
Age at diagnosis*	0.70	0.52–0.94	0.017	1.03	0.94–1.13	0.513	1.04	0.77–1.39	0.815
Duration of NET									
Up to 8 weeks	Ref			Ref			Ref		
> 8 to 24 weeks	1.55	0.53–4.55	0.425	1.05	0.82–1.34	0.723	0.84	0.37–1.92	0.682
> 24 weeks	3.50	1.28–9.59	0.015	0.91	0.69–1.19	0.478	0.99	0.42–2.33	0.972
Both ER and PR positive									
No	Ref			Ref			Ref		
Yes	1.68	0.58–4.81	0.338	0.78	0.61–1.00	0.050	0.59	0.28–1.24	0.163
Histology									
Ductal only	Ref			Ref			Ref		
Lobular only	1.44	0.42–4.92	0.566	2.38	1.67–3.39	< 0.001	2.41	0.88–6.57	0.087
Ductal and lobular	1.02	0.40–2.58	0.975	2.63	2.07–3.35	< 0.001	1.53	0.68–3.44	0.306
Other	2.07	0.76–5.64	0.156	1.51	1.03–2.21	0.037	0.82	0.19–3.56	0.791
Clinical stage group**									
I	Ref			Ref			Ref		
II	0.56	0.27–1.14	0.111	0.44	0.36–0.55	0.000	4.58	1.05–19.97	0.043
III	–			0.18	0.11–0.31	0.000	21.30	4.85–93.52	< 0.001

BAA women more likely to downstage to pT0/is than White women (OR 2.9, CI 1.2–6.7), as well as those who received > 24 weeks of NET compared to < 8 weeks of treatment (OR 3.5, CI 1.3–9.6)

*OR* odds ratio, *CI* confidence interval, *BAA* Black/African American, *ref* reference group, *NET* neoadjuvant endocrine therapy, *ER* estrogen receptor, *PR* progesterone receptor

*Variable was transformed to interpret results as the odds of outcome associated with a 10-year increase in age at diagnosis

**None of the clinical Stage III tumors downstaged to pT0/is, so this category was not included in this model

## Discussion

This investigation confirms racial disparities in HR+ breast cancer presentation, while also highlighting the limitations of the current therapeutic strategy in overcoming this disparity. Independent of NET duration and clinical stage at presentation, BAA women were more likely to experience both complete tumor response and progression to metastatic disease. Our results indicate additional and previously unreported complexity of unpacking drivers of disparate outcomes in BAA and White women with HR+ breast cancer.

In selecting the women to be included in the study, we noted greater attrition in the eligible BAA female population due to HR− disease, a known contributor to breast cancer mortality disparities between BAA and White women [10]. Of those with HR+ breast cancer, a greater percentage of BAA women compared to White women received neoadjuvant chemotherapy. HER2-positive disease is often managed with neoadjuvant chemotherapy and may explain part of the observed difference; however, across races, there was a similar rate of HER2 positivity. Thus, this difference may be indicative of higher stage at presentation or more aggressive clinicopathologic features, such as Ki67, among BAA women that warrant more aggressive upfront intervention. Indeed, rates of the more aggressive HR+ subtype, luminal B disease, are more prevalent in BAA women [11].

Furthermore, among the cohort who received NET, BAA women were more likely to present with more advanced disease and higher co-morbidity index. They were also more likely to have Medicaid insurance and reside in ZIP codes with lower household income and lower education level; these are known contributors to later stage presentation among BAA women [12]. Our study demonstrates that BAA women were more likely to live in an urban area and receive treatment at an academic or NCI-designated comprehensive cancer center (CCC). Most CCCs are concentrated in urban areas. Urban areas have higher incidence of pollution and PM2.5 [13]; there is evidence that these SDHs contribute to worse biology [14]. Recent studies demonstrate the triple-negative subtype to be associated with food deserts as well as air pollution [15]. SDHs have not yet been studied in relation to lower ER expression among HR+ breast cancer, and even less on their impact on endocrine therapy resistance. The interplay of SDH and therapy resistance offers a potential area of breast cancer disparity research.

Still, studies demonstrate that mortality differences persist even when controlling for SDH and access to care. Indeed, BAA women with HR+ breast cancer enrolled on clinical trials where access is provided and stage at presentation and disease type are controlled continue to have higher rates of resistance to therapy as measured by recurrence [16, 17]. Furthermore, as demonstrated by Benefield et al., compared to White women, BAA women with HR+ breast cancer with similar insurance, co-morbidity indices, education ,and location had persistently worse outcomes [18]. Thus, differences in therapy resistance across races might contribute to the observed disparities in mortality.

Therapy resistance is a common problem in HR+ breast cancer. Compared to 40–50% in triple-negative and 60–70% in HER2-enriched phenotypes, the pathologic complete response rate after neoadjuvant chemotherapy in HR+ breast cancer is 10–13% [19]. Because chemotherapy is not universally effective, the mainstay treatment is endocrine therapy. Five years of adjuvant treatment has shown benefit of reducing both recurrence and death [20]. However, despite its administration, many women with early-stage HR+ breast cancer will recur [21]. Thus, there has been some focus on tailoring adjuvant endocrine therapy duration to mitigate recurrence rates. As several investigations have demonstrated, women with more advanced disease, such as node-positive disease, may benefit from longer duration of endocrine therapy. Thus, 10 years of endocrine therapy has been utilized in such women based on presenting clinical findings. However, many of those studies had small sample sizes and were unable to distinguish a racial difference in response [16, 22]. Furthermore, there are currently no established biomarkers that predict development of endocrine resistance. In contrast to giving endocrine therapy in the adjuvant setting, offering endocrine therapy in the neoadjuvant setting allows observation of endocrine resistance with the tumor in situ. Studies have shown NET to be as effective as neoadjuvant chemotherapy in HR+ breast cancer [23].

Our study evaluates de novo endocrine resistance across racial groups by evaluating tumor and nodal response to NET. Certain characteristics in both groups were associated with tumor-upstaging rates, such as clinical stage. Additionally, lobular carcinoma had a twofold higher risk of upstaging compared to invasive ductal carcinoma. Lobular carcinoma is traditionally endocrine sensitive as it is more often associated with high-hormone receptor positivity. However, lobular carcinoma is also notoriously under-appreciated on traditional imaging modalities, including mammogram and ultrasound [24]. Tumor size by magnetic resonance imaging (MRI) was not included in this evaluation and may correct for a degree of the tumor upstaging rate seen. Because of the nuances of imaging of lobular breast cancer, it is difficult to ascertain the true effect of endocrine therapy on upstaging rates in this population. Further work is necessary to assess a comparison of clinical T stage on MRI with final staging in the setting of NET.

When comparing racial groups, BAA women had a higher percentage of receipt of NET. BAA women were also, on average, treated for longer duration of time. It is unclear why BAA women were more likely to receive longer durations of endocrine therapy. This may be due to the blunted response witnessed, necessitating a longer duration of therapy to achieve a response. Additionally, socioeconomic factors may also have played a role. Studies have demonstrated increased length of time between biopsy and initiation of surgical management in BAA women compared to NHW women with similar insurance status [25, 26]. Previous studies investigating duration of therapy included few BAA women (mean 2%) [22]. Delays in surgical intervention among BAA women may have contributed to an increased length of time between initiation of therapy and surgery.

Even when controlling for higher grade/stage at presentation, histology, age, ER and PR positivity, and longer duration of NET, BAA women were still more likely to be pT0/is and M1. This may indicate greater baseline endocrine resistance that may be overcome with longer duration of therapy for tumors that eventually respond and downstage. However, the differential outcomes to treatment also suggest that the baseline endocrine resistance among BAA women can only be overcome at earlier stage presentation, with later stage presentation more likely to progress to metastatic disease. Further research is needed to understand the change in molecular expression over the time course of a tumor.

There are several limitations with this study that pertain to the retrospective nature of database review. For example, NCDB does not provide the ER and PR percentages for participants. Given that response to endocrine therapy is in part due to the hormone responsiveness of the tumor, such information would give further insight into the differences in biology between the races. Furthermore, the basis for determinations of clinical stage was not defined; therefore, differences between races pertaining to access to care and availability of advanced imaging modalities, such as tomosynthesis and MRI, are unknown. Additionally, while the start date of endocrine therapy was captured, adherence to therapy during the neoadjuvant period was not. A previous study led by Wheeler et al. of 1280 women (43.2% BAA) demonstrated that despite similar durations of adjuvant endocrine therapy, BAA women compared to White women had lower rates of adherence during the study period [27]. A similar study has not been conducted in the neoadjuvant setting, and it is unclear if the nonadherence reported in studies translates to the neoadjuvant setting when endocrine therapy is given for a much shorter duration with a defined endpoint. Further prospective work is needed to control for these potential confounders and examine the effect of endocrine therapy in the neoadjuvant period across races.

Nonetheless, this study offers important insight into the differences in HR+ breast cancer biology across races and, importantly, about response to treatment. It also begins to investigate the complex interplay of social determinants of health, race, tumor biology, and therapy resistance. We observed more aggressive tumor biology among BAA women with HR+ breast cancer but especially at higher stage of disease. Given the large disparity in mortality from HR+ breast cancer between BAA and White women and the complexity of race, more research is needed to identify drivers of this disparity as well as therapies to mitigate this. There is undoubtedly a great deal of heterogeneity in the population that is not fully explored in this analysis; future studies should work to understand the influence of population heterogeneity on disease presentation. Furthermore, biomarker development may offer more insight into detecting endocrine resistance upfront, especially in BAA women who may derive less benefit from endocrine therapy alone.

## Data Availability

The datasets analyzed during the current study are publicly available in the National Cancer Database repository.
